# Supplementary material for: High dietary intake of unsaturated fatty acids is associated with improved insulin resistance – a cross-sectional study based on the NHANES database
Source: Lipids Health Dis. 2023 Dec 5;22:216. doi: 10.1186/s12944-023-01982-1 (PMC10696799; doi:10.1186/s12944-023-01982-1)
Supplement: Supplementary file 1 — Supplementary Material 1 [file 12944_2023_1982_MOESM1_ESM.pdf]

1698908396

By Cross Check

WORD COUNT 5773

TIME SUBMITTED 02-NOV-2023 02:59AM

PAPER ID 103926284

High dietary intake of unsaturated fatty acids is associated with improved insulin resistance – a cross-sectional study based on the NHANES database

Abstract:

Background: A moderate intake of unsaturated fatty acids (UFA) is associated positively with improved insulin resistance. The aim of this study was to investigate the relationship between the dietary intake of unsaturated fatty acids/total fats (UFA/TF) and insulin resistance.

Methods: 15,560 participants were selected from the National Health and Nutrition Examination Survey (NHANES) database enrolled between March 2017 and 2020, and excluded those under 20 years of age, pregnant, or with missing data for key research items. Finally, 7,630 participants were included in the study. R software was used for data analysis that included: 1) general descriptive statistics; 2) comparison of differences in baseline information of three UFA/TF groups, namely low, medium, and high ratios; 3) calculation of the correlation between the UFA/TF ratio and markers of insulin resistance: triglyceride-glucose index (TyG) and homeostatic model assessment for insulin resistance (HOMA-IR); 4) stratification of the study subjects into two groups, with or without insulin resistance, using a cut-off value of HOMA-IR ≥2, followed by logistic regression analysis to examine the relationship between UFA/TF and insulin resistance status in the two groups; and 5) further stratification of the subjects according to age, gender, body mass index (BMI), race, total energy intake, total protein, total carbohydrate, total sugars, total dietary fiber, total fat, alcohol consumption, diabetes, hypercholesterolemia to analyze the impact of UFA/TF on insulin resistance status in different subgroups.

Results: 1) **A high UFA/TF level was associated with a low TyG index and HOMA-IR [β (vs. TyG index) = -0.559, 95% *CI*: (-0.821~-0.297), *P*<0.001; β (vs. HOMA-IR) = -0.742, 95% *CI*: (-1.083~-0.402), *P*<0.001].** This negative relationship became more pronounced when UFA/TF exceeded 57.9% (i.e., the higher group). 2) **Logistic regression analysis showed that a higher UFA/TF level was associated with a lower risk of developing insulin resistance [Q3 vs Q1: 0.838 (95%*CI*: 0.709~0.991); *P* for trend=0.038].** After adjusting for covariates such as gender, age, and BMI, this protective effect remained significant **(*P* value < 0.05)**. 3) Analysis also showed that increased UFA/TF intake reduced the risk of developing insulin resistance

(*OR*=0.266, 95% *CI*: (0.075~0.946), *P*=0.041). Subgroup analysis showed that although elevated UFA/TF intake showed no statistically significant difference in its effect in most subgroups, the large study population in this study provides valuable insights on potential changes. Increased UFA/TF intake may confer relatively greater benefits within specific subgroups, particularly among the elderly [Q3 age group, *OR*=0.114, 95%*CI*: (0.012~1.078), *P*=0.058], females [*OR*=0.234, 95%*CI*: (0.041~1.333), *P*=0.102], those with a BMI ≤ 25 kg/m^2 [*OR*=0.191, 95%*CI*: (0.016~2.344), *P*=0.196], and individuals without hypercholesterolemia [*OR*=0.207, 95%*CI*: (0.042~1.013), *P*=0.0519]. The impact of high UFA/TF levels within subgroups based on the presence or absence of coronary heart disease and stroke displayed contrasting trends. In those without coronary heart disease, there was a significant protective effect against insulin resistance [*OR*=0.254, 95% *CI*: (0.07~0.929), *P*=0.0384], while in the stroke subgroup, a significantly protective effect against insulin resistance was observed [*OR*=0.002, 95%*CI*: (0~0.695), *P*=0.0376].

Conclusion: A high dietary intake of UFA relative to total fat consumption **could be** a protective factor against the risk of developing insulin resistance.

Keywords: **unsaturated dietary fats; dietary formulations; insulin resistance;** triglyceride-glucose index; HOMA-IR

Introduction

Improvements in the standard of living and dietary patterns have resulted in aggregation of the components of metabolic disorders and an increase in clinical symptoms such as central obesity, blood lipid disorders, glucose metabolism disorders, hypertension, and vascular diseases. The etiology of the metabolic syndrome has received increasing attention in recent years. Insulin resistance is now regarded as the central link in the mechanism for development of these metabolic disorders and is often associated with factors such as a long-term lack of exercise, excessive energy intake, and unhealthy dietary patterns. UFA is an important component of body fat and include monounsaturated and polyunsaturated fatty acids that are derived from foods such as vegetables, fruits, nuts, and fish. *In vitro* [1, 2] and *in vivo* [3, 4] studies have shown that UFA improve insulin sensitivity by regulating metabolic-related

cellular pathways. Several cohort studies [5-7] have also reported that a moderate intake of UFA improves insulin resistance. However, most of these studies were small-scale and conducted in single centers, lacked broad, cross-sectional data and did not thoroughly investigate how varying intake levels of UFA related to insulin resistance. We hypothesize that a change in the dietary pattern of lipid nutrients may reduce insulin resistance and thus improve metabolic disorders. The current study therefore analyzed data from the National Health and Nutrition Examination Survey (NHANES) to examine this relationship using the UFA/total fat ratio (UFA/TF) and established markers of insulin resistance. The study also compared the impact of different dietary UFA ratios on the risk of developing insulin resistance, and investigated the role of UFA/TF in different subgroups of subjects stratified according to gender, age, body mass index (BMI), and presence of baseline diseases. This analysis allowed us to examine in detail the relationship between the proportion of UFA intake in the diet and insulin resistance.

Methods

Study population

NHANES is a cross-sectional survey of the American population, which combines self-reported survey data and physical examination data to measure the prevalence of major diseases and risk factors. The data in the NHANES database are divided into unrestricted public data and limited access data. Under the NHANES Data Release and Access Policy (https://www.cdc.gov/nchs/data/nhanes/nhanes_release_policy.pdf), researchers have free access to unrestricted public data without the need for a license. In this analysis, NHANES unrestricted public data from 2017 to March 2020 has been used [8], collected prior to the coronavirus disease 2019 (COVID-19) pandemic, that included a total of 15,560 participants. Individuals aged ≥20 years were included and pregnant women and subjects without data on blood lipids, fasting blood glucose, insulin, total fat intake, and UFA intake were excluded (Figure 1). 7,630 participants were finally analyzed using these selection criteria. The study was approved by the Institutional Review Board of the Centers for Disease Control and Prevention in the United States, and all participants signed informed consent forms.

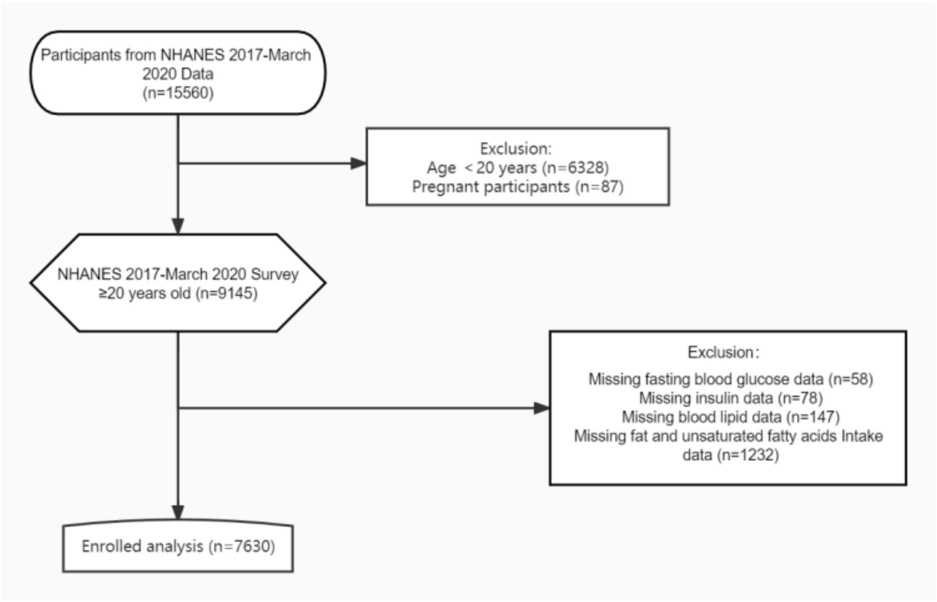

Figure 1 The participant recruitment process for the experiment

Independent variable

The independent variable used in this study was the UFA/TF ratio. The formula for calculating this variable is: The sum of total monounsaturated fatty acids (MUFA) and polyunsaturated fatty acids (PUFA) divided by **total** fatty acids (i.e., **the sum of saturated fatty acids (SFA), MUFA, and PUFA. The UFA/TF values were then divided into three groups based on size, with Q1: $0 \leq Q1 < 0.552$, $X \pm S = 0.501 \pm 0.044$; Q2: $0.552 \leq Q2 < 0.612$, $X \pm S = 0.582 \pm 0.017$; and Q3: $0.612 \leq Q3 \leq 0.849$, $X \pm S = 0.657 \pm 0.035$.** Data regarding dietary intake were obtained from the data subset, "Dietary Data," that represented two 24-hour diet recalls that assessed the consumption of total mono and polyunsaturated and saturated fat. The first dietary recall was conducted at the Mobile Examination Center (MEC), while the second recall took place via telephone between 3 to 10 days after the MEC visit. The dietary intake data allowed estimation of the type and quantity of food and beverages (including all types of fluid consumed in the 24 hours prior to the recall interviews). This information was then used to calculate the consumption of energy, nutrients, and other food components. A set of measuring tools (glasses, bowls, mugs, bottles, spoons, measuring cups, rulers, thickness sticks, bean bags, and circles) was provided in the dietary interview room to aid with the reports on food

portions (NHANES measuring guides). After the in-person dietary interview, the participants received measuring cups, spoons, a ruler, and a food model booklet containing 2D drawings of the measuring tools. These drawings were used as an aid during the telephone interviews. Dietary data from the 2017-March 2020 pre-pandemic sample were processed using USDA's Food and Nutrient Database for Dietary Studies (FNDDS) 2017-2018. FNDDS 2019-2020 was used for intakes reported from 2019-March 2020, and then merged with the NHANES 2017-2018 data. The FNDDS contains comprehensive information on coded foods, portion sizes, and nutrient values. To account for potential differences in nutrient and calorie intake between weekdays and weekends, ratio-adjusted weights were applied to ensure the sample more accurately reflected the general intake levels of the participants **[9].**

Outcome variables

The following three indicators were used as outcome variables in the study: HOMA-IR index, triglyceride-glucose (TyG) index, and insulin resistance status. HOMA-IR was calculated as fasting insulin (μU/mL) x fasting blood glucose (mmol/L) ÷ 22.5, while the TyG index was calculated as the natural logarithm of fasting triglycerides (mg/dL) x fasting blood glucose (mg/dL) ÷ 2. Based on the results of previous studies [10-14] insulin resistance was defined as a HOMA-IR value ≥ 2.0. The participants were classified into two groups according to their insulin resistance status. The laboratory test results used to calculate these outcome variables were obtained from the "Laboratory Data" subset.

Covariates

Several covariates were considered in this study, including sex, age, race, BMI, intakes of **total energy intake, total protein, total carbohydrate, total sugar, total dietary fiber, and total fat,** alcohol consumption, diabetes, and hypercholesterolemia. Demographic variables (sex, age, race) were obtained from the "Demographic Variables" data subset, BMI from the "Body Measures" subset, and total energy and nutrient intakes from the "Dietary Data" subset. Information on alcohol consumption, diabetes, hypertension, and hypercholesterolemia were obtained from the "Medical Conditions" questionnaire subset. Blood pressure was measured three times and averaged to determine hypertension status. A participant was considered hypertensive **[15]** if their mean systolic blood pressure was ≥ 130 mmHg, or mean diastolic

blood pressure was greater ≥ 80 mmHg in the "Blood Pressure" data subset. Smoking status was not included as a covariate because of a large amount of missing data.

Statistical analysis

The data analyses for this study followed the guidelines of the official website of the the United States Centers for Disease Control (US CDC: https://wwwn.cdc.gov/nchs/nhanes/tutorials/default.aspx). The study participants were categorized into three groups based on their UFA/TF status; low, medium, or high. Differences in the baseline data of the three groups were compared. Normally distributed continuous variables were expressed as mean ± standard deviation, variables with a skewed distribution as median (interquartile range, IQR), and categorical variables as n (%). The Mann-Whitney U test (continuous variables) and Chi-square test (categorical variables) were used to examine statistical differences between the different groups. The mean differences among the three quantile groups were further examined using Tukey post-hoc analysis. Then the study participants were categorized into two groups based on their insulin resistance status, i.e., presence or absence of insulin resistance. Logistic regression analysis and inflection point analysis was used to determine the association between the UFA/TF ratio and insulin resistance status. Finally, the participants were stratified based on gender (male or female), age (tertiles), BMI (≤ 25 or >25 kg/m^2) [16], and baseline disease conditions (presence of hyperlipidemia or cardiovascular disease). The impact of the UFA/TF ratio on insulin resistance status in these different subgroups was then analyzed. All the statistical analyses were conducted using R software, version 4.2.2 (R Foundation for Statistical Computing, Vienna, Austria), with P-values < 0.05 considered statistically significant.

Results

Baseline characteristics of the subjects

As shown in Table 1, the study included 3745 males and 3885 females with a mean age of 50.95 ± 17.34 years. Compared to patients in the Q1 and Q2 groups, the patients in the Q3 group had a higher mean age and higher high-density lipoprotein (HDL) levels, but lower mean values of BMI, waist-to-height ratio (WtHR), and levels of insulin, fasting blood glucose,

triglycerides, total cholesterol (CHO), and low-density lipoprotein (LDL). The differences in BMI, WtHR, and levels of insulin, fasting blood glucose, triglycerides, LDL, and HDL were statistically significant between the three groups. For smoking and alcohol consumption, the smoking rate was significantly higher in the Q1 and Q2 groups than in the Q3 group, although there was no significant difference in alcohol consumption between the three groups. There was also no significant difference in the presence of baseline diseases between the three groups. However, there were significant differences in total daily energy and macronutrient (carbohydrate, fat, protein) intake between the three groups. Differences in insulin resistance markers among the groups were further compared, including HOMA-IR and TyG levels. As shown in Table 1,higher levels of UFA/TF are associated with lower insulin resistance index (HOMA-IR) and TyG index , especially in the Q3 group, which had significantly lower levels compared to the other two groups.

Table 1. Baseline characteristics of the subjects

**Variables**	**Total (n = 7630)**	**UFA/TF ratio Q1 (n = 2541)**	**UFA/TF ratio Q2 (n = 2546)**	**UFA/TF ratio Q3 (n = 2543)**	***P* value**
**Gender, n (%)**					0.030
Male	3745 (49.1)	1280 (50.4)	1271 (49.9)	1194 (47)	
Female	3885 (50.9)	1261 (49.6)	1275 (50.1)	1349 (53)	
**Age**	50.95±17.34	50.3±17.83	50.39±17.20	52.11±16.93	<0.001
**Race, n (%)**					<0.001
Mexican American	877 (11.5)	311 (12.2)	305 (12)	261 (10.3)	
Other Hispanic	772 (10.1)	276 (10.9)	265 (10.4)	231 (9.1)	
Non-Hispanic white	2737 (35.9)	1126 (44.3)	887 (34.8)	724 (28.5)	
Non-Hispanic black	2046 (26.8)	501 (19.7)	707 (27.8)	838 (33)	
Other Race (Including multi-racial)	1198 (15.7)	327 (12.9)	382 (15)	489 (19.2)	
**BMI**	30.12 ± 7.58	30.02 ± 7.37	30.51 ± 7.73	29.83 ± 7.62	0.005

**WHtR**	0.61 ± 0.10	0.61 ± 0.10	0.61 ± 0.11	0.60 ± 0.10	0.015
**Energy (kcal)**	2039.14 ± 865.06	2057.55 ± 902.47	2097.96 ± 864.36	1961.85 ± 821.14	<0.001
**Carbohydrate (g)**	438.78 ± 217.54	445.07 ± 231.67	458.22 ± 218.86	413.03 ± 198.41	< 0.001
**Sugars (g)**	187.42 ± 124.88	202.23 ± 138.97	194.81 ± 123.70	165.21 ± 106.86	< 0.001
**Dietary fiber (g)**	30.17 ± 18.18	27.39 ± 16.51	30.47 ± 17.21	32.66 ± 20.22	< 0.001
**Protein (g)**	144.62 ± 70.84	145.31 ± 73.02	152.16 ± 71.12	136.38 ± 67.41	< 0.001
**Total fat (g)**	154.70 ± 82.10	151.43 ± 82.33	161.90 ± 81.58	150.76 ± 81.93	< 0.001
**Total saturated fatty acids (g)**	49.34 ± 28.90	58.14 ± 33.09	51.30 ± 26.81	38.59 ± 22.25	< 0.001
**Total unsaturated fatty acids (g)**	89.83 ± 49.12	76.41 ± 42.29	94.30 ± 47.62	98.76 ± 53.90	< 0.001
**Insulin (μU/mL)**	14.98 ± 24.22	15.54 ± 23.55	15.82 ± 27.84	13.55 ± 20.61	0.047
**Fasting glucose (mmol/L)**	6.31 ± 2.12	6.33 ± 2.26	6.34 ± 2.13	6.26 ± 1.95	0.545
**Triglyceride (mmol/L)**	1.24 ± 1.09	1.30 ± 1.24	1.28 ± 0.96	1.15 ± 1.05	0.001
**Total cholesterol (mmol/L)**	4.80 ± 1.05	4.82 ± 1.05	4.80 ± 1.05	4.78 ± 1.06	0.348
**HDL cholesterol (mmol/L)**	1.38 ± 0.42	1.36 ± 0.41	1.36 ± 0.40	1.42 ± 0.43	<0.001
**LDL cholesterol (mmol/L)**	2.83 ± 0.92	2.86 ± 0.95	2.87 ± 0.89	2.76 ± 0.93	0.012
**Current smoking, n (%)**					<0.001
No	1849 (57.0)	631 (53.7)	589 (55)	629 (63)	
Yes	1396 (43.0)	545 (46.3)	482 (45)	369 (37)	
**Alcohol drinking, n (%)**					0.439
No	658 (8.9)	204 (8.3)	230 (9.3)	224 (9.1)	
Yes	6761 (91.1)	2260 (91.7)	2254 (90.7)	2247 (90.9)	
**Diabetes, n (%)**					0.064
No	6441 (84.4)	2176 (85.7)	2147 (84.4)	2118 (83.3)	
Yes	1187 (15.6)	364 (14.3)	398 (15.6)	425 (16.7)	
**Hypercholesteremia, n (%)**					0.592
No	4820 (63.6)	1619 (64.2)	1614 (63.8)	1587 (62.9)	
Yes	2756 (36.4)	903 (35.8)	915 (36.2)	938 (37.1)	
**Coronary artery disease, n (%)**					0.375

No	7264 (95.50)	2406 (95.14)	2435 (95.94)	2423 (95.43)	
Yes	342 (4.50)	123 (4.86)	103 (4.06)	116 (4.57)	
**Stroke, n (%)**					0.812
No	7223 (94.83)	2399 (94.64)	2412 (94.81)	2412 (95.04)	
Yes	394 (5.17)	136 (5.36)	132 (5.19)	126 (4.96)	
**HOMA-IR, median (IQR)**	2.66 (1.56, 4.80)	2.78 (1.63, 4.89)	2.75 (1.63, 5.03)	2.42(1.46, 4.47)	0.002
**TyG index**	8.68 ± 0.64	8.72 ± 0.65	8.72 ± 0.64	8.61 ± 0.62	<0.001

Linear relationship between UFA/TF and the insulin resistance index

As shown in Figures 2A and 2B, higher levels of UFA/TF are associated with lower levels of TyG and HOMA-IR indicators.. This negative correlation persisted after adjustment for covariates and was statistically significant. In the adjusted model (Figure 2C), UFA/TF in Model 1 correlated negatively with insulin resistance [β (vs. TyG index) = -0.678, 95% *CI:* (-0.953~-0.403), P<0.001; β (vs. HOMA-IR) = -0.715, 95% *CI*: (-1.057~-0.373), *P*<0.001], with this correlation remaining after excluding the influences of total energy and nutrient intakes, alcohol consumption, and presence of metabolic diseases [β (vs. TyG index) = -0.436, 95% *CI*: (-0.704~-0.168), *P=0.001*; β (vs. HOMA-IR) = -4.855, 95% *CI*: (-9.114~-0.595), *P=0.026*]. The inflection point analysis also showed that the negative correlation with TyG became more apparent when UFA/TF exceeded 0.579, while the negative correlation with HOMA-IR became more apparent when UFA/TF exceeded 0.626.

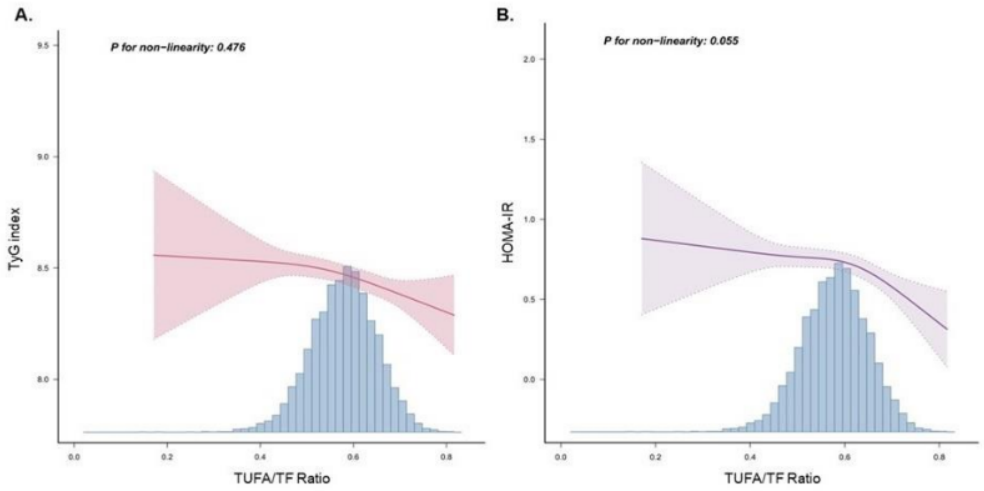

C.

	Crude, *P* value	M1, *P* value	M2, *P* value
**HOMR-IR**	-0.646 (-1.056,-0.235), *0.002*	-0.715 (-1.057~-0.373), *<0.001*	-4.855 (-9.114~-0.595), *0.026*
**TyG**	-0.59 (-0.884,-0.296), *< 0.001*	-0.678 (-0.953~-0.403), *<0.001*	-0.436 (-0.704~-0.168), *0.001*

Note: The crude analysis did not include the effects of covariables; M1 (Model 1) represents the analysis after adjusting for the influence of the following covariables: age, gender, BMI; M2 (Model 2) represents the analysis adjusted for the effects of the following covariates: age, gender, BMI, race, total energy intake, total protein, total carbohydrate, total sugars, total dietary fiber, total fat, alcohol consumption, diabetes, hypercholesterolemia.

Figure 2 The linear relationship between UFA/TF and insulin resistance index

The relationship between different levels of dietary UFA/TF and insulin resistance

Logistic regression analysis was used to examine the relationship between different levels of dietary UFA/TF and the risk of insulin resistance. As shown in Table 2, no significant difference was observed in the risk of IR between subjects with medium or low levels of dietary UFA/TF [ORQ2 vs Q1=1.059; 95%CI: (0.895~1.253); P for trend=0.507]. But when the level of UFA/TF in the diet was high (group Q3), the risk of IR was reduced significantly compared to that in subjects with a low dietary ratio [$OR_{Q3\ vs\ Q1}$=0.838; 95%*CI*: (0.709~0.991); *P* for trend=0.038]. This protective effect persisted after adjustment for covariates in Models 1 and 2, with the trends remaining statistically significant ($p < 0.05$). Aithough this significant difference weakened under the more comprehensive correction used in model 2.

Table 2 Logistic analysis of the relationship between different levels of dietary UFA/TF and insulin resistance status

	UFA/TF ratio Q1	UFA/TF ratio Q2	UFA/TF ratio Q3
Crude OR (95% *CI*), *P* value	Ref	1.059 (0.895~1.253), *0.507*	0.838 (0.709~0.991), *0.038*
M1 OR (95% *CI*), *P* value	Ref	0.970 (0.797~1.180), *0.758*	0.785 (0.645~0.956), *0.016*
M2 OR (95% *CI*), *P* value	Ref	0.977 (0.794~1.203), *0.828*	0.805 (0.648~1), *0.049*

Note: The crude analysis did not include the effects of covariable. M1 (Model 1) represents the analysis after adjusting for the influence of the following covariables: age, gender, and BMI. M2 (Model 2) represents the analysis adjusted for the effects of the following covariates: age, gender, BMI, race, total energy intake, total protein, total carbohydrate, total sugars, total dietary fiber, total fat, alcohol consumption, diabetes, and hypercholesterolemia.

Relationship between dietary UFA/TF and insulin resistance status in the different subgroups

For the subgroup analysis, the study participants were stratified according to age, gender, BMI, race, total energy intake, total protein, total carbohydrate, total sugars, total dietary fiber, total fat, alcohol consumption, diabetes, and hypercholesterolemia. Specifically, the participants were categorized by age into Q1 (30.72±6.326 yr), Q2 (51.47±5.586 yr), and Q3 (70.77±6.686 yr). As shown in Figure 3, There was a significant correlation between increased UFA/TF intake and a reduced risk of insulin resistance ("Total" subgroup, *P=0.041*). Further subgroup analysis showed that although elevated UFA/TF intake showed no statistically significant differences in its effect across most subgroups, the large study population in this study added valuable insights on these relationships. An increased UFA/TF intake may confer relatively greater benefits within specific subgroups, particularly among the elderly (Q3 age group), females, those with a BMI ≤ 25 kg/m^2, and individuals without hypercholesterolemia. Notably, the impact of high UFA/TF levels within subgroups based on the presence or absence of coronary heart disease and stroke displayed contrasting trends. In individuals without coronary heart disease, there was a significant protective effect against insulin resistance [*OR*=0.254; 95% *CI*: (0.07~0.929); *P*=0.0384], while in the stroke subgroup, a significantly protective effect on insulin resistance was observed [*OR*=0.002; 95% *CI*:(0~0.695); *P*=0.0376]. This disparity may be attributable to potential confounding factors influencing study outcomes or actual differences in the effects of UFA on cardiovascular and

cerebrovascular health. It is suggested that further specialized studies are warranted to investigate these findings. Nevertheless, on the whole, higher levels of UFA/TF intake have varying degrees of a protective effect against insulin resistance in different subgroups .

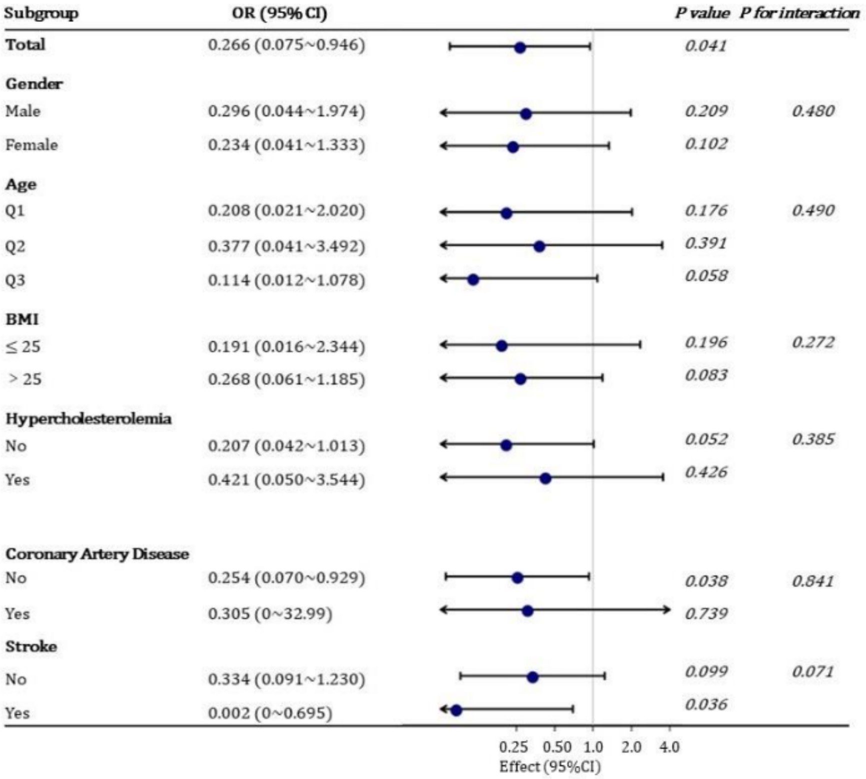

Note: The subgroup analysis was adjusted for the effects of the following covariates: age, gender, BMI, race, total energy intake, total protein, total carbohydrate, total sugars, total dietary fiber, total fat, alcohol consumption, diabetes, and hypercholesterolemia. Logistic regression model subgroup analysis was used with a statistical significance level of $p<0.05$.

Figure 3 The relationship between dietary UFA/TF and insulin resistance in different subgroups

Discussion

The metabolic syndrome is characterized by the development of several metabolic disorders and mainly results from insulin resistance. The syndrome increases the risk of developing and dying from diabetes, dyslipidemia, or cardiovascular diseases, and has become a global crisis in clinical and public health. This study used NHANES data to investigate the relationship

between the dietary intake of different proportions of UFA and the risk of developing insulin resistance. The baseline data showed that this population had a high prevalence of overweight and obesity, with 68.9% classified as overweight and 31.1% as obese, raising the risk of chronic conditions like cardiovascular diseases and diabetes. In addition, their diet included excessive intake of fat and sugar, along with unhealthy lifestyle choices such as a 43.0% smoking rate and 91.1% alcohol consumption. It is well known that poor dietary structure and unhealthy eating habits lead to excessive fat intake, high total energy, and an energy imbalance, resulting in a series of metabolic disorders. A post-hoc analysis was used to compare the mean values of various parameters in quartile groups of the UFA/TF ratio (Q1, Q2, Q3) and showed significant differences between these groups. Individuals in Q3 had lower BMI and waist-to-hip ratio, suggesting a potentially reduced susceptibility to obesity-related diseases. Conversely, Q2 had higher daily energy and carbohydrate intakes, possibly increasing the risk of developing a metabolic disorder. Elevated triglyceride levels in Q2 also indicated a slightly higher risk of cardiovascular disease risk. In addition a higher median HOMA-IR index in Q2 indicated a certain degree of insulin resistance. These preliminary observations provide initial clues for further investigations into the risk of developing various diseases. Therefore, people at high risk of metabolic disorders should be protected through dietary intervention. Currently, relevant dietary guidelines emphasize the types and proportions of fats required in a healthy diet. In addition to reducing the total amount of fat, the proportion of each type of fatty acid intake also needs to be balanced [17]. Fatty acids are an effective biomarker of dietary fat intake, with their measurement now used as a supplementary tool in nutritional epidemiology surveys to monitor fat intake and also study the development of various diseases.

The insulin resistance index, HOMA-IR and the triglyceride glucose index, TyG have been shown to be reliable and direct markers for evaluating insulin resistance, and are important for diagnosing and assessing the prognosis of metabolic-related diseases. For example, a survey of elderly patients with diabetes showed an association of HOMA-IR and TyG indices with metabolic disorders, oxidative stress, and increased risk of cardiovascular and metabolic-related diseases [18]. There is evidence that HOMA-IR is also an independent risk factor for myocardial glucose uptake disorders [19], while the TyG index predicts the incidence of future cardiovascular adverse events in patients with diabetes complicated by acute coronary

syndrome, independent of other known cardiovascular risk factors [20-22]. Similarly, a recent survey of healthy subjects reported that a greater intake of UFA and vegetables in the diet reduced fasting insulin levels and HOMA-IR [23]. Our study showed that when the proportion of UFA intake in the diet was higher than half of the total fat intake, HOMA-IR and TyG levels decreased and showed a negative correlation with the UFA/TF ratio. Therefore, the subjects were further divided into two groups using a cut-off level of HOMA-IR ≥ 2.0 and showed that a high proportion of dietary UFA could the risk of insulin resistance.

MUFA are found primarily in olive oil, avocados, nuts, and seeds, while PUFA are sourced mainly from fatty fish, flaxseeds, chia seeds, walnuts, and vegetable oils such as sunflower and corn oil. Most MUFA and n-3 PUFA, such as alpha-linolenic acid (ALA), eicosapentaenoic acid (EPA), and docosahexaenoic acid (DHA) have a protective effect on human metabolism and physiological processes, including the inflammatory response [24]. International studies have suggested that a high intake of PUFA affects glucose and lipid metabolism, anti-inflammatory responses, and immune regulation, thereby reducing the risk of cancer [25,26]. Miyamoto and coworkers [27] reported that PUFA affected energy regulation in obese mice and reduced their body weight, CHO, insulin resistance, and glucose tolerance to levels lower than that measured in obese mice eating a regular diet. Furthermore, population-based studies have shown that dietary interventions that change fatty acid intake and composition reduce the risk of developing type 2 diabetes [28]. In a double-blind clinical trial, Liu et al. [29] evaluated the different effects of n-3 PUFA on glucose and lipid metabolism in patients with diabetes. After a six-month follow-up, patients in the intervention group who received additional n-3 PUFA showed significant reductions in insulin and C-peptide levels. Patients in this treatment group also had significant decreases in CHO, apolipoprotein A1, and interleukin-6 levels [29]. In our study, the results also demonstrated that individuals with a higher proportion of UFA in their diet had a lower BMI and WtHR, higher levels of HDL, and lower levels of triglycerides, cholesterol, and LDL. In terms of blood glucose metabolism, their insulin and blood glucose levels were lower than those who consumed fewer UFA. These results therefore confirm earlier studies that reported increasing the proportion of UFA in the diet helped optimize human glucose and lipid metabolism.

Abnormal blood lipid metabolism is a significant risk factor for atherosclerosis, while insulin resistance causes increased vascular fibrosis and stiffness that results in the progression of cardiovascular diseases [30]. On the other hand, increased levels of plasma free fatty acids promote insulin resistance, further exacerbating beta-oxidation of fatty acids and leading to a vicious cycle [31-33]. Therefore, reducing blood lipid levels, especially non-high-density lipoprotein-cholesterol (non-HDL-C), and correcting insulin resistance are essential goals for controlling atherosclerosis and preventing coronary heart disease and stroke. While both MUFA and PUFA improve lipid profiles, their relevance to clinical cardiovascular events appears to be somewhat dissimilar [34, 35]. Evidence supporting the cardiovascular benefits of MUFA remain relatively limited [36]. In addition, in terms of improving glycemic control, substituting PUFA for carbohydrate or SFA intake is associated with enhanced insulin secretion, reduced fasting blood glucose, and lowered glycosylated hemoglobin (HbA1c). In contrast, when compared to PUFA, while replacing carbohydrate or SFA intake with MUFA shows a trend towards improved fasting blood glucose and HbA1c, these changes are not statistically significant [37]. Furthermore, for anti-inflammatory properties, although MUFA activate beneficial anti-inflammatory mechanisms, thereby reducing chronic inflammation and subsequently improving overall metabolism, elevated MUFA levels in the body do not consistently have a positive impact on inflammation. For example, it has been shown in patients with chronic kidney disease that an increased MUFA/SFA ratio in blood lipids is associated with elevated circulating levels of CRP, suggesting the potential for exacerbation of inflammation. The Reduction of Cardiovascular Events with Icosapent EthylIntervention Trial (REDUCE-IT) [39], a large randomized controlled trial also investigated the impact of n-3 PUFA on the occurrence of cardiovascular events. The results showed a 25% reduction in the risk of major cardiovascular events and a 20% reduction in the risk of cardiovascular death. Another randomized controlled trial [40] also reported that dietary alpha linolenic acid (ALA) lowered the levels of CHO, LDL, and triglycerides, while also exhibiting anti-inflammatory effects. Tindall et al. [41] used a sample population to assess the impact on established cardiovascular risk factors of adjusting the proportion of UFA in the diet whilst maintaining a constant daily caloric intake. Their results suggested that substitution of saturated fatty acids with monounsaturated and PUFA in the diet significantly reduced already established

cardiovascular risk factors, including triglycerides, LDL, and non-HDL-C. However, the study also pointed out that after six weeks of UFA substitution therapy, there was no improvement in the extent of arterial hardening in the study subjects. Similarly, the statin residual risk with epanova in high cardiovascular risk patients with hypertriglyceridemia (STRENGTH) randomized clinical trial conducted by Nicholls et al. [42] showed that additional intake of n-3 PUFA in combination with standard background therapy did not have a significant impact on the composite outcome of primary adverse cardiovascular events. **The current study suggested that a higher proportion of UFA intake had a protective effect against the development of insulin resistance in the general population, and that this protective effect was particularly evident in females, older individuals, and those without hypercholesterolemia or coronary artery disease. While increasing UFA/TF intake did not exhibit statistically significant differences in its effects across most subgroups, higher levels of UFA/TF did have a positive impact by improving insulin resistance within the overall population. This effect remained consistent regardless of the participants' gender, age, daily energy and macronutrient intake, or underlying medical conditions. For individuals at high risk of developing insulin resistance, in addition to controlling the total daily caloric intake and the proportion of carbohydrates, total fat, and total protein in their diets, increasing the proportion of UFA in their fat consumption may also had a positive effect.**

Strengths and limitations

In the past, dietary recommendations for improving insulin resistance and metabolic disorders have often focused only on controlling the total intake of nutrients. The results of the current study suggest that the balance of fatty acids consumed is also important for improving metabolic disorders. For patients who have difficulty strictly controlling their dietary intake, adjusting the proportion of nutrients initially and then gradually achieving a controlled dietary intake plan may help to improve compliance, thereby promoting a continuous improvement in insulin resistance.

There were several limitations in this study. Different types of fatty acids have different biological effects in the human body. Previous studies [43] have shown that n-3 PUFA rich in fish oil induce the production of pro-resolving lipid mediators that have anti-inflammatory

properties targeted at cardiovascular risk. In contrast, n-6 PUFA primarily produce thromboxane A2, thereby promoting atherosclerosis and thrombus formation. Another study [44] has shown that n-6 PUFA may increase insulin sensitivity, whereas n-3 PUFA do not. Although we compared the relationship between the intake of total UFA in the diet and the level of insulin resistance, we did not carry out further investigations on different types of fatty acids. **Studies [45, 46] have shown that smoking is related to insulin resistance and metabolic abnormalities, although due to the lack of data, smoking status was not included as a covariate in this study. This represents another limitation of the study.** In addition, as the primary use of the NHANES database is to evaluate the health and nutritional status of the U.S. population, the sample population in this study was comprised mainly of White and Black individuals, with a proportionally smaller representation of other ethnic groups. Therefore, relevant studies and comprehensive evaluations of populations in other countries and regions are required to validate our findings.

Conclusions

The results of this study suggest that a higher level of dietary intake of unsaturated fats is associated with lower blood lipid and glucose levels, and that increasing the proportion of dietary unsaturated fat intake may improve glucose and lipid metabolism. Based on these results, we suggest that higher levels of unsaturated fat in the diet may be a protective factor associated with a lower risk of developing insulin resistance. **This study provides informations for clinical practice on on more effective dietary interventions for improving insulin resistance in high-risk populations. For individuals who may find it challenging to strictly manage their caloric and macronutrient intake from the outset, initiating a dietary intervention plan that emphasizes increasing the proportion of UFA-rich foods in the diet to alleviate insulin resistance and subsequently improve metabolic disturbances is a worthwhile consideration.**

1698908396

ORIGINALITY REPORT

21%

SIMILARITY INDEX

PRIMARY SOURCES

1	www.ncbi.nlm.nih.gov<br>Internet	144 words — 2%
2	www.researchsquare.com<br>Internet	114 words — 2%
3	www.mdpi.com<br>Internet	71 words — 1%
4	wwwn.cdc.gov<br>Internet	50 words — 1%
5	journals.plos.org<br>Internet	37 words — 1%
6	www.science.gov<br>Internet	37 words — 1%
7	"Biomarkers in Cardiovascular Disease", Springer Science and Business Media LLC, 2016<br>Crossref	31 words — 1%
8	www.researchgate.net<br>Internet	31 words — 1%
9	Wenqiang Che, Yujiao Wang, Xiangyu Wang, Jun Lyu. "Midlife brain metastases in the United States: Is male at risk?", Cancer Medicine, 2022<br>Crossref	30 words — 1%

10 Toh, Eu-Wing, Peter Brown, Eva Morris, Ian Botterill, and Philip Quirke. "Area of Submucosal Invasion and Width of Invasion Predicts Lymph Node Metastasis in pT1 Colorectal Cancers :", Diseases of the Colon & Rectum, 2015.
Crossref

28 words — < 1%

11 worldwidescience.org
Internet

25 words — < 1%

12 www.locus.ufv.br
Internet

25 words — < 1%

13 Mann, Jim, Truswell, Stewart, Hodson, Leanne. "Essentials of Human Nutrition 6e", Essentials of Human Nutrition 6e, 2023
Publications

24 words — < 1%

14 Toshiyuki Nakanishi, Yoshiki Sento, Yuji Kamimura, Kazuya Sobue. "Macintosh laryngoscope, i-view™, and C-MAC® video laryngoscopes for tracheal intubation with an aerosol box: A randomized, crossover, manikin study", Research Square Platform LLC, 2020
Crossref Posted Content

23 words — < 1%

15 www.frontiersin.org
Internet

21 words — < 1%

16 apjcn.nhri.org.tw
Internet

20 words — < 1%

17 article.imrpress.com
Internet

20 words — < 1%

18 www.nature.com
Internet

19 words — < 1%

19	wwwnc.cdc.gov<br>Internet	19 words — < 1%
20	Teresa M. Seccia, Gian Paolo Rossi. "Clinical Use and Pathogenetic Basis of Laboratory Tests for the Evaluation of Primary Arterial Hypertension", Critical Reviews in Clinical Laboratory Sciences, 2008<br>Crossref	17 words — < 1%
21	research.library.mun.ca<br>Internet	17 words — < 1%
22	stacks.cdc.gov<br>Internet	17 words — < 1%
23	www.foodandnutritionresearch.net<br>Internet	17 words — < 1%
24	www.spandidos-publications.com<br>Internet	17 words — < 1%
25	Antonio Corsello, Chiara Maria Trovato, Elisabetta Di Profio, Sabrina Cardile et al. "Ketogenic diet in children and adolescents: The effects on growth and nutritional status", Pharmacological Research, 2023<br>Crossref	16 words — < 1%
26	Joan A. Vaccaro, Joanne Niego, Fatma G. Huffman. "Dietary factors, body weight, and screen time in U.S. children with and without asthma", Children's Health Care, 2014<br>Crossref	16 words — < 1%
27	bmccardiovascdisord.biomedcentral.com<br>Internet	16 words — < 1%

28 Philip C. Calder, Richard J. Deckelbaum. "Editorial: Omega-3 fatty acids: new studies, new data, new questions", Current Opinion in Clinical Nutrition & Metabolic Care, 2021 — 15 words — < 1%
Crossref

29 Shengtao Liu, Lanhui Yu, Zhiyuan Lu, Chiwen Cheng, Xuejun Gu, Jingying Liu, Xingtao Zhou. "Effect of keratometric astigmatism on visual outcomes following small incision lenticule extraction", Frontiers in Medicine, 2022 — 15 words — < 1%
Crossref

30 Shutang Zhang, Jie Tian, Min Lei, Canye Zhong, Yan Zhang. "Association between dietary fiber intake and atherosclerotic cardiovascular disease risk in adults: a cross-sectional study of 14,947 population based on the National Health and Nutrition Examination Surveys", BMC Public Health, 2022 — 15 words — < 1%
Crossref

31 ijponline.biomedcentral.com — 14 words — < 1%
Internet

32 John A. Colwell. "Aspirin Therapy in Diabetes is Underutilized", Diabetes Care, 2001 — 13 words — < 1%
Crossref

33 www.scirp.org — 13 words — < 1%
Internet

34 Jindan Luo, Xin Xu. "Dietary fiber intake and the risk of bladder cancer in the Prostate, Lung, Colorectal and Ovarian (PLCO) cohort", Carcinogenesis, 2020 — 12 words — < 1%
Crossref

35 Mohammad Mohammadi, Azadeh Nadjarzadeh, Masoud Mirzaei, Hossien Fallahzadeh et al. "Dietary glycemic index and glycemic load in association with sleep duration: YaHS-TAMYZ and Shahedieh observational studies", Clinical Nutrition ESPEN, 2021
Crossref
12 words — < 1%

36 S Shin, J Sung, H Joung. "A fruit, milk and whole grain dietary pattern is positively associated with bone mineral density in Korean healthy adults", European Journal of Clinical Nutrition, 2014
Crossref
12 words — < 1%

37 Stephanie M George, Yikyung Park, Michael F Leitzmann, Neal D Freedman et al. "Fruit and vegetable intake and risk of cancer: a prospective cohort study", The American Journal of Clinical Nutrition, 2009
Crossref
12 words — < 1%

38 Yonghui Liu, Juan Chen, Shangchu Wang, Xihong Zhou. "Effects of two concentrations of dietary tribasic zinc sulfate on growth performance, gut morphology, and zinc transporter expression levels in pigs", Animal Biotechnology, 2022
Crossref
12 words — < 1%

39 Shichao Sun, Pingye Wang. "The association between e-moped usage and residents' subjective well-being: a case study of Shanghai, China by using Bayesian network", Transportation Planning and Technology, 2023
Crossref
11 words — < 1%

40 etd.uwc.ac.za
Internet
11 words — < 1%

41 www.researcherslinks.com

Internet

11 words — < 1%

42 Yvonne J. H. Janssen, Frans Helmerhorst, Marijke Frölich, Ferdinand Roelfsema. "A Switch from Oral (2 mg/Day) to Transdermal (50 µg/Day) 17β-Estradiol Therapy Increases Serum Insulin-Like Growth Factor-I Levels in Recombinant Human Growth Hormone (GH)-Substituted Women with GH Deficiency", The Journal of Clinical Endocrinology & Metabolism, 2000
Crossref

10 words — < 1%

43 academic.oup.com
Internet

10 words — < 1%

44 researchonline.jcu.edu.au
Internet

10 words — < 1%

45 www.medrxiv.org
Internet

9 words — < 1%

46 G. H. Meng, D. Song, L. B. Li, C. J. Yang, Z. X. Qu, Y. P. Gao. "Dietary methionine requirement of Jing Brown layer hens from 9 to 17weeks of age", Journal of Animal Physiology and Animal Nutrition, 2017
Crossref

8 words — < 1%

47 K MASUO, T KATSUYA, T OGIHARA, M TUCK. "Acute hyperinsulinemia reduces plasma leptin levels in insulin-sensitive Japanese men", American Journal of Hypertension, 2005
Crossref

8 words — < 1%

48 Kalyan R. Chitturi, Bhaskar Bhardwaj, Ghulam Murtaza, Poorna R. Karuparthi et al. "Clinical impact of tricuspid regurgitation on transcatheter edge-to-

8 words — < 1%

edge mitral valve repair for mitral regurgitation", Cardiovascular Revascularization Medicine, 2022
Crossref

49	assets.researchsquare.com<br>Internet	8 words — < 1%
50	dspace.uef.fi<br>Internet	8 words — < 1%
51	groups.google.com<br>Internet	8 words — < 1%
52	iraqjournals.com<br>Internet	8 words — < 1%
53	link.springer.com<br>Internet	8 words — < 1%
54	livrepository.liverpool.ac.uk<br>Internet	8 words — < 1%
55	synapse.koreamed.org<br>Internet	8 words — < 1%
56	www.ajcn.org<br>Internet	8 words — < 1%
57	Jing Li, Mengzi Sun, Xuhan Wang, Fengdan Wang, Yibo Dong, Jiaxin Fang, Zechun Xie, Xiaotong Li, Ruirui Guo, Yuxiang Wang, Bo Li. "Association of Physical Activity with Phenotypic Age: Among Population with Different Breakfast Habits", Research Square Platform LLC, 2023<br>Crossref Posted Content	7 words — < 1%
58	Bisher Sawaf, Sarya Swed, Hidar Alibrahim, Haidara Bohsas et al. "Triglyceride-Glucose Index	6 words — < 1%

as Predictor for Hypertension, CHD and STROKE Risk among non-Diabetic patients: A NHANES Cross-Sectional Study 2001-2020", Research Square Platform LLC, 2023
Crossref Posted Content

EXCLUDE QUOTES	OFF	EXCLUDE SOURCES	OFF
EXCLUDE BIBLIOGRAPHY	ON	EXCLUDE MATCHES	OFF
